# Mitigating secondary salinization in grapes: long-term benefits of biochar and cow dung

**DOI:** 10.3389/fpls.2025.1528354

**Published:** 2025-02-28

**Authors:** Hongyin Zhou, Zhong Yu, Shiying Zhang, Qinghou Zong, Yulian Zhang, Yuhan Pang, Naiming Zhang, Xianrong Yue, Yishu Deng, Yunsheng Xia

**Affiliations:** ^1^ College of Resources and Environment, Yunnan Agricultural University, Kunming, China; ^2^ Yunnan Soil Fertilization and Pollution Remediation Engineering Research Center, Yunnan Agricultural University, Kunming, China; ^3^ Bio-Big Data Intelligent Application Center, Huazhi Biotechnology Co. Ltd, Changsha, China; ^4^ College of Architecture and Engineering, Yunnan Agricultural University, Kunming, China

**Keywords:** biochar, cow dung, secondary salinization, grapes, antioxidant oxidase

## Abstract

Secondary salinization of soil seriously hinders the healthy cultivation of facility grapes. Biochar has been shown to mitigate the negative effects of saline stress on plants. However, the long-term response mechanism between the soil’s key physicochemical properties, ion concentration, and enzyme activity and the physiological resistance of facility grape plants to biochar combined with cow dung application to alleviate the soil secondary salinization stress remains unclear. In this study, a field experiment was set up once in September 2021 with five different treatments, including no amendments. which was used as the blank control (CK), and application of biochar (10 t·ha^-1^, T1), cow dung (30 t·ha^-1^, T2), biochar mixed with cow dung (5 t·ha^-1^+15 t·ha^-1^, T3), and biochar mixed with cow dung (10 t·ha^-1^+30 t·ha^-1^, T4), respectively. The results showed that compared with the CK treatment, application treatments significantly reduced soil total salt(TS) content and the electrical conductivity(EC) value; increased soil water-stable aggregates and nutrient content; stimulated an increase in soil urease (S-UE), sucrose (S-SC) and phosphatase(S-ALP)activities; and changed soil exchangeable calcium and magnesium ion concentrations. Among the treatments, the T4 treatment reduced TS and EC by 73.03% and 61.11%, respectively. Biochar combined with cow dung significantly increased chlorophyll content and reduced malondialdehyde content (MDA), the activities of superoxide dismutase (SOD), peroxidase (POD), and catalase (CAT) in grape leaves. The T4 treatment decreased MDA, SOD, POD, and CAT by 54.59%, 40.14%, 44.28%, and 70.17% compared with the CK treatment, respectively. Correlation analysis showed that the balance of soil exchangeable calcium and magnesium ions and the stability of soil aggregate structure were the key factors in alleviating soil secondary salinization stress. In conclusion, biochar combined with cow dung application can alleviate the oxidative stress response of grape plants and improve the quality of grapes by improving the structure of soil water-stable aggregates, coordinating the concentration of soil exchangeable calcium and magnesium ions, and stimulating soil enzyme activity.

## Graphical Abstract

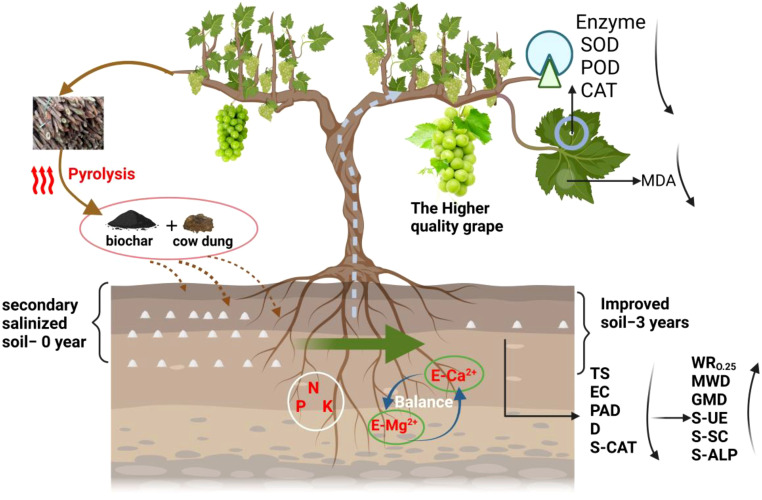

## 1 Introduction

Grape (*Vitis vinifera* L.) is a cash crop grown globally with high economic and nutritional value (Dong et al., 2023; Zhang et al., 2023b). Among these, the Shine Muscat grape is deeply appreciated by people because of its strong rose fragrance, high sugar and low acid content, neat and beautiful ears, and other characteristics. According to statistics, the global planting area of Shine Muscat grape is as high as 6.7 × 10^4^ ha (Li et al., 2023; Ren et al., 2024). The development of the grape industry has brought huge economic benefits to farmers. However, the secondary salinization of the facility soil results in the deterioration of soil quality after years of high-intensity planting, excessive fertilization, and unreasonable production management, severely affecting the healthy development of the grape industry. Soil secondary salinization is caused by the intensification of agricultural production and is a form of soil degradation (Hu et al., 2020; Cai et al., 2022). Secondary salinization leads to soil compaction and imbalance of crop nutrient supply, destroys the normal physical and chemical properties of soil, and directly affects the growth and development of crops. Therefore, developing effective measures to alleviate the secondary salinization of grape facilities is of practical significance.

Biochar is a carbon-rich residue produced from agricultural and forestry waste such as straw in the temperature range of 300°C to 1000°C under anaerobic or anoxic conditions (Sun et al., 2020). It has carbon fixation, emission reduction, and sink enhancement functions. Thus, the technology of biochar preparation by biomass pyrolysis has been promoted and applied worldwide, and many types and huge quantities of agricultural and forestry wastes are generated globally every year. The comprehensive utilization of biochar is of great significance for waste resource utilization (Zhou et al., 2024). Biochar, as a soil amendment, has positive effects in terms of improving soil physicochemical properties and plant growth (Duan et al., 2021b). Studies have shown that the application of biochar in maize cultivation enhanced soil nutrient content, thereby increasing maize growth and yield (Liu et al., 2021). In addition, biochar was able to increase the pH and organic carbon content of rice soils, thereby enhancing rice yield and nutrient content (Khan et al., 2021). Studies on potatoes have also found a significant increase in yield and tuber size with the application of biochar (Akhtar et al., 2015). In addition, the application of biochar in fruit tree cultivation (e.g., apple and citrus trees) can effectively improve soil fertility and water retention capacity, thus enhancing fruit tree growth and fruit quality (Liu et al., 2022). Biochar is also effective in amending degraded soils and mitigating soil salinization (Zhang et al., 2023a). However, the application of biochar also has different effects. For example, biochar leads to enhanced salt migration and promotes salt leaching and accumulation in arid areas due to its own characteristics (Feng et al., 2020). In addition, the application of organic fertilizers can also improve soil fertility and minimize plant toxicity caused by secondary salinization (Liang et al., 2005). However, the continuous application of organic fertilizers such as animal manure can also lead to an increase in soluble salt content in the soil, aggravating the potential risk of soil secondary salinization (Yao et al., 2007).

At present, the effect of biochar and organic fertilizers in improving secondary salinized soil and improving crop quality is limited to a single basic study. Furthermore, the mechanism of the oxidative stress response to biochar and organic fertilizers in improving plant stress resistance remains unclear. Although some relevant studies have focused on the combined application of biochar and organic fertilizers on degraded soil in facilities, they are still in the stage of pot exploration or indoor culture research and lack medium to long-term field verification. Moreover, biochar mixed with different raw materials has different effects on various crops with different stress factors. Therefore, this study applied grape straw biochar and cow dung alone or in combination. It aimed to investigate the long-term improvement effect of grape straw biochar combined with farm manure on secondary salinized soil and grape quality and explore the physiological mechanism of resistance to abiotic stress. The results will help promote the effective utilization of waste biomass resources and residual nutrients in soils and provide a theoretical and application basis for improving soil and grape quality.

The aim of the study was to determine (1) whether the long-term effect of biochar combined with cow dung on reducing soil total salt (TS) content and electrical conductivity (EC) is better than that of single application; (2) whether biochar and cow dung will synergistically improve soil nutrient conditions, aggregates stability and enzyme activities; and (3) whether biochar and cow dung will synergistically alleviate the degree of membrane lipid peroxidation, and the antioxidant stress response of leaves to improve grape quality through improving soil quality.

## 2 Materials and methods

### 2.1 Study location

The study was conducted in Binchuan County, Dali City, Yunnan Province, China, in the dry-hot valley area on the south bank of the Jinsha River in China and at the edge of Yunling Hengduan Mountain Range. Binchuan is a low-latitude plateau with a dry winter and wet summer in the middle tropics. The average annual temperature is 17.9°C, and the annual rainfall is 559.4 mm. The climate is extremely suitable for crop growth. In 2023, the facility grape planting area in Binchuan County reached 12,200 ha, the fruit planting area was 11,900 ha, the annual output was 462,900 t, and the total production value exceeded 4.619 billion yuan. This improvement experiment was conducted in a greenhouse in the Jipingguan grape growing area. The daytime and nighttime temperatures in the greenhouse were 28 ± 3°C and 20 ± 2°C, respectively, with a photoperiod of 13 h. The greenhouse-cultivated soil undergoes severe secondary salinization, and the process of salinization is caused mainly by unreasonable man-made measures such as excessive application of fertilizers, unreasonable irrigation, and large evaporation capacity because of the facility’s cultivation conditions. This is prevalent globally, especially in areas with developed irrigated agriculture. The problem of soil salinization in Binchuan County epitomizes the global challenge of soil salinization. According to the Food and Agriculture Organisation of the United Nations (FAO, 2021), approximately 833×10^6^ ha of soil is threatened by salinization globally, mainly in Eurasia, Africa, and the western United States of America. Therefore, this study on the improvement of salinized soil in Binchuan County is important for understanding the dynamics, impacts, and solutions to global soil salinization and its results could be useful for other regions.

According to the Chinese soil nutrient grading standard, the test soil was highly salinized with a nutrient imbalance, including EC value > 1500 µS·cm^-1^ and TS content > 1000 mg·kg^−1^. The basic physical and chemical properties of the tested soil are shown in **Table 1**.

**Table 1:** Basic physical and chemical properties of the tested soil.

pH	EC (µS·cm^-1^)	TS (mg·kg^−1)^	OM (g·kg^−1^)	AP (mg·kg^−1^)	AK (mg·kg^−1^)	NN (mg·kg^−1^)	Soil texture (%)		
							Grit	Powder	Cosmid
7.78	2823	1716	21.62	4.92	313	341	25.12	33.05	38.20

OM, organic matter; AP, available phosphorus; AK, available potassium; NN, nitrate nitrogen.

### 2.2 Study material

The tested biochar was grape straw biochar (pyrolyzed at 500°C under anoxic conditions, with a pH value of 9.67 and a total carbon content of 455 g·kg^-1^) to promote the utilization of waste resources and returning grape straw to the field soil. The tested farm manure was fermented and decomposed cow dung (total nutrient content is not less than 45%, N, P, K nutrient ratio is 15∶15∶15), which is a traditional bio-organic fertilizer commonly used in the local area. The tested biochar and cow dung were purchased from Yunnan Nongjiale Agricultural Group Co., Ltd., Kunming, China.

The grape cultivar used in the study was *Sunshine Rose*.

### 2.3 Study design

This improvement experiment was carried out on the basis of the previous experimental results from an indoor culture improvement experiment on secondary saline soils in a grape-producing area. The indoor culture improvement experiment was conducted with eight different treatments (biochar, peat, cow dung, bagasse organic fertilizer, biochar and cow dung, biochar and bagasse organic fertilizer, peat and cow dung, and peat and bagasse organic fertilizer), and among these, the biochar and cow dung combined treatment was selected for the medium to long-term effect verification test in a field experiment because it had the best improvement effects.

This improvement experiment was conducted in September 2021. A total of five treatments were set up in the study: CK: no amendments (blank control); T1: biochar (10 t·ha^-1^); T2: cow dung (30 t·ha^-1^); T3: biochar (5 t·ha^-1^) + cow dung (15 t·ha^-1^); and T4: biochar (10 t·ha^-1^) + cow dung (30 t·ha^-1^). Each treatment was repeated three times, with a total of 15 plots. The area of each plot was 2 m × 2.5 m = 5 m^2^. The spacing between plots was 1 m, and the spacing for vine planting was 1 m × 2.8 m. Amendments of different treatments were added to both sides of the vines in each plot, which were evenly mixed with 0–30 cm soil tilling. The management measures for the viticulture period were consistent with those for other viticultures in the same greenhouse.

### 2.4 Sample collection and analysis

#### 2.4.1 Sample collection

Soil, grape leaves, and fruit samples were collected in July 2024 during the grape ripening stage. Five soil subsamples were randomly collected from each plot from the 0–30 cm soil layer, excluding subsoil layers, using the diagonal method with the grape tree as the center line and mixed into a representative soil sample for each treatment. After the samples were brought back to the laboratory, the soil was gently stripped into small pieces along the natural structure, and the plant roots and small stones were removed. An appropriate amount of fresh soil was taken from each treatment and stored in a refrigerator at − 20°C. Other soil samples were naturally air-dried and passed through 2 mm, 1 mm, and 0.149 mm sieves for testing and analysis. Soil aggregates were measured by dry and wet sieve methods. At the same time, the upper leaves of each treatment were collected, sealed in a foam box with dry ice, brought back to the laboratory, and immediately stored in a freezer at − 80°C for use. Finally, four representative fruit ears and 20 fruits in the ripening stage were randomly selected from each treatment group, collected in a foam box filled with dry ice, and sealed and stored in a refrigerator at –20°C for quality determination.

#### 2.4.2 Determination of soil aggregate stability

Aggregates were measured by a TPF-100 aggregate structure analyzer. The upper and lower swing frequency of the sieve was set to 30 times/min, and the time was set to 10 min. Aggregates with different particle sizes were obtained, transferred to an aluminum box, and dried at 105°C. The evaluation indexes of soil aggregate stability included average weight diameter (MWD)(mm), geometric mean diameter (GMD) (mm), non-water stable aggregate content (DR_0.25_) (%), and water stable aggregate content (WR_0.25_) (%). Aggregates with particle size > 0.25 mm are called soil aggregate structures, which have good structural stability, water stability, and nutrient retention (Wang et al., 2023a). The contents of non-water-stable and water-stable aggregates in undisturbed soil were obtained using a dry sieve and a wet sieve. Larger MWD and GMD values indicate more stable agglomerates (Liu et al., 2024). Soil fractal dimension (D) is a parameter reflecting the geometric shape of soil structure. The smaller the fractal dimension of particle size distribution of aggregate structure, the better the stability of soil aggregates (Cao et al., 2021).

The calculation formula is as follows:


(1) $$MWD=\sum_{i=1}^{n}({\bar{x}}_{i}{\text{w}}_{i})$$

(2) $$GMD=exp[\frac{\sum_{i=1}^{n}w_{i}ln\;{\bar{x}}_{i}}{\sum_{i=1}^{n}w_{i}}]$$

(3) $$DR_{0.25}=\frac{M_{DR>0.25}}{M_{D}}$$

(4) $$WR_{0.25}=\frac{M_{WR>0.25}}{M_{W}}$$

(5) $${\bar{x}}_{i}=(x_{i}+\;x_{i-1})/2$$

In the above **Equations 1**–**5**: ${\bar{x}}_{i}\;$ is the average diameter (mm) of i grain size; ${\bar{x}}_{i}$ represents the aperture (mm) of the i-th sieve, *x_0_* = *x_1_*, *x_n_* = *x_n+1_*; ${\bar{x}}_{i}$ is the percentage (%) of the aggregate mass in the i particle size interval to the total mass of the aggregate; *M_DR>0.25_* and *M_WR>0.25_* are the particle mass (g) of the dry sieve and wet sieve aggregates with particle size > 0.25 mm, respectively. *MD* and *MW* are the total mass (g) of dry sieve and wet sieve aggregates, respectively.

The soil fractal dimension (D) is calculated according to the following formula:


(6) $$\frac{M(\delta \;<\;{\bar{x}}_{i})}{M}+{\left(\frac{{\bar{x}}_{i}}{{\bar{x}}_{max}}\right)}^{3-D}$$

In the formula: (δ < ${\bar{x}}_{i}$ ) represents the total mass of aggregates with particle size less than ${\bar{x}}_{i}$ , and ${\bar{x}}_{max}$ is the average particle size of the largest particle size of aggregates; after taking the logarithm of both sides of Equation 6, the score shape dimension D is calculated by regression analysis.

#### 2.4.3 Determination of soil chemical properties

The pH value of the soil was determined by the potentiometric method, and the soil-to-water ratio was 2.5:1. The soil TS content was measured by mass method and EC was determined using a soil-to-water ratio of 1:5 using an FE30 Mettler conductivity meter. The content of organic matter (OM) was determined by the K_2_Cr_2_O_7_-H_2_SO_4_ and FeSO_4_ volumetric methods. The content of available phosphorus (AP) was determined by sodium bicarbonate extraction and the molybdenum-antimony resistance colorimetric method. The content of available potassium (AK) was extracted using ammonium acetate and determined using a flame meter. The contents of exchangeable calcium ion (E-Ca^2+^) and exchangeable magnesium ion (E-Mg^2+^) were determined by atomic absorption spectrophotometry. The content of nitrate nitrogen (NN) was determined using a flow injection analyzer.

#### 2.4.4 Determination of soil enzyme activity

The change in absorbance of the solution after reacting with the soil was measured using an ultraviolet photometer to determine the activity of catalase (S-CAT). Urease (S-UE) activity was measured by employing indophenol blue colorimetry with urea as the substrate. A visible light photometer was utilized to quantify the activity of sucrase (S-SC), which facilitated the degradation of sucrose by reacting with 3,5, 1–2-nitrosalicylic acid to generate brown-red compounds. The activity of alkaline phosphatase (S-ALP) was determined with a visible spectrophotometer to quantify the amount of phenol produced during the hydrolysis of disodium phenyl phosphate to phenol.

#### 2.4.5 Determination of grape fruit quality

The content of reducing sugar (RS) in all frozen grape fruit was determined by anthrone colorimetry after thawing at room temperature (Xia et al., 2013). For this, a 0.5 g sample was weighed and ground to a homogenate. Next, 10 mL of 80% ethanol was added and the sample was boiled in a water bath at 80°C for 40 min and centrifuged at 4,000 rpm for 10 min. Activated carbon was added to the supernatant for 30 min and filtered. Further, 0.2 mL of the sample liquid was absorbed, 0.8 mL of distilled water and 5 mL of anthracone reagent were added, the mixture was boiled in a water bath for 10 min, and the light absorption value at 625 nm was determined using a spectrophotometer. The sugar content was obtained from the standard curve (anthrone, glucose, and distilled water) and expressed in g·L^-1^. The dissolved solid (DS) content was determined using a PAL^-1^ digital display saccharometer after extracting juice from each treated grape fruit and expressed as a percentage. The vitamin C (VC) content was quantitatively determined using 2,6-dichlorophenol indophenol titration (da Silva et al., 2017). Next, a 10 g sample was weighed and ground after adding 5 mL of 2% oxalic acid solution. After filtration, 10 mL of the filtrate was titrated with the calibrated 2,6-dichlorophenol indophenol solution until a pink color developed. The VC content was calculated according to the dosage volume. It was expressed in mg·100 g^-1^. The organic acid (TA) content was determined by the acid-base titration method. Further, 5 g of the sample was weighed, ground with distilled water (30 mL) to a homogenate, washed at 80°C for 30 min, and filtered to a volume of 50 mL. Then, 10 mL of the sample solution was titrated with 0.1 mol·L^-1^ sodium hydroxide standard solution and three to five drops of phenolphthalein were added until a red color developed. The organic acid content was calculated according to the volume consumed and expressed in g·L^-1^.

#### 2.4.6 Determination of malondialdehyde and chlorophyll content in grape leaves

The malondialdehyde (MDA) content was determined by the method proposed by Zhou et al (Zhou et al., 2023). Briefly, the samples (0.1 g) were homogenized in 1 mL of 0.1% (w/v) trichloroacetic acid (TCA). The homogenate was centrifuged at 16,000 g and 4°C for 10 min. A 500 -μL aliquot of the supernatant was mixed with an equal amount of 0.5% thiobarbituric acid in 20% TCA. The mixture was incubated at 95°C for 30 min, cooled on ice, and centrifuged at 12,000 g for 5 min. The absorbance of the supernatant was measured at 532 nm, and the non-specific absorption was measured at 600 nm. The MDA content was calculated from the extinction coefficient of 155 mM^-1^ cm^-1^ using the equation C = [Abs (535 – 600) ÷ 155,000] x 10^6^. The MDA content was expressed as nmol MDA g^−1^ fresh weight. The chlorophyll (CHL) content in the upper leaves of each treatment was measured by a Minolta SPAD-502 at the mature stage.

#### 2.4.7 Determination of antioxidant enzyme activity in grape leaves

For the determination of CAT activity, frozen grape leaves (0.5 g) were ground in a mortar containing a phosphate buffer solution (pH 7.0). The samples were centrifuged at 17,500 g and 4°C for 15 min. The supernatant (100 µL) was mixed with 1.9 mL of phosphate buffer (pH 7.0) and 0.01% H_2_O_2_ (1 mL). The enzyme activity was measured at 240 nm using an ultraviolet-visible (UV-vis) spectrophotometer (Implen P300, Duren, Germany) (Iwaniuk et al., 2022). The CAT activity was expressed as U·mg^-1^ protein·min^-1^. For the determination of POD activity, frozen grape leaves (0.5 g) were ground in a mortar containing phosphate buffer solution (pH 7.0), and the samples were centrifuged at 17,500 g and 4°C for 15 min. The supernatant (100 µL) was mixed with 2.7 mL of phosphate buffer (pH 7.0), followed by the addition of 0.04% MnCl2 (20 µL) and 0.14% NADH (150 µL) solutions. The enzyme activity was measured at 340 nm using a UV-vis spectrophotometer (Implen P300) (Iwaniuk et al., 2022). The POD activity was expressed as U·mg^-1^ protein·min^-1^. For the determination of SOD activity, frozen grape leaves (0.5 g) were ground in a mortar containing a phosphate buffer solution (pH 7.8). The samples were centrifuged at 17,500 g and 4°C for 15 min. The supernatant (50 µL) was mixed with 2.2 mL of phosphate buffer (pH 7.8) and 0.006% riboflavin (250 µL), followed by the addition of 0.0023% methionine (250 µL) and 0.06% NBT (250 µL) solutions. The enzyme activity was measured at 560 nm using a UV-vis spectrophotometer (Implen P300) (Iwaniuk et al., 2022). The SOD activity was expressed as U·mg^-1^ protein.

### 2.5 Statistical analysis

Microsoft Excel 2019 was used to organize the data and calculate the mean value and standard error. The SPSS 27.0 software was used for statistical analysis of the data, Origin 2021 software was used to draw bar charts, and correlation plots were used as correlation analysis charts. Duncan’s test was used to evaluate the significant differences between different treatments (*P* < 0.05).

## 3 Results

### 3.1 Soil TS and EC values

**Figure 1:**
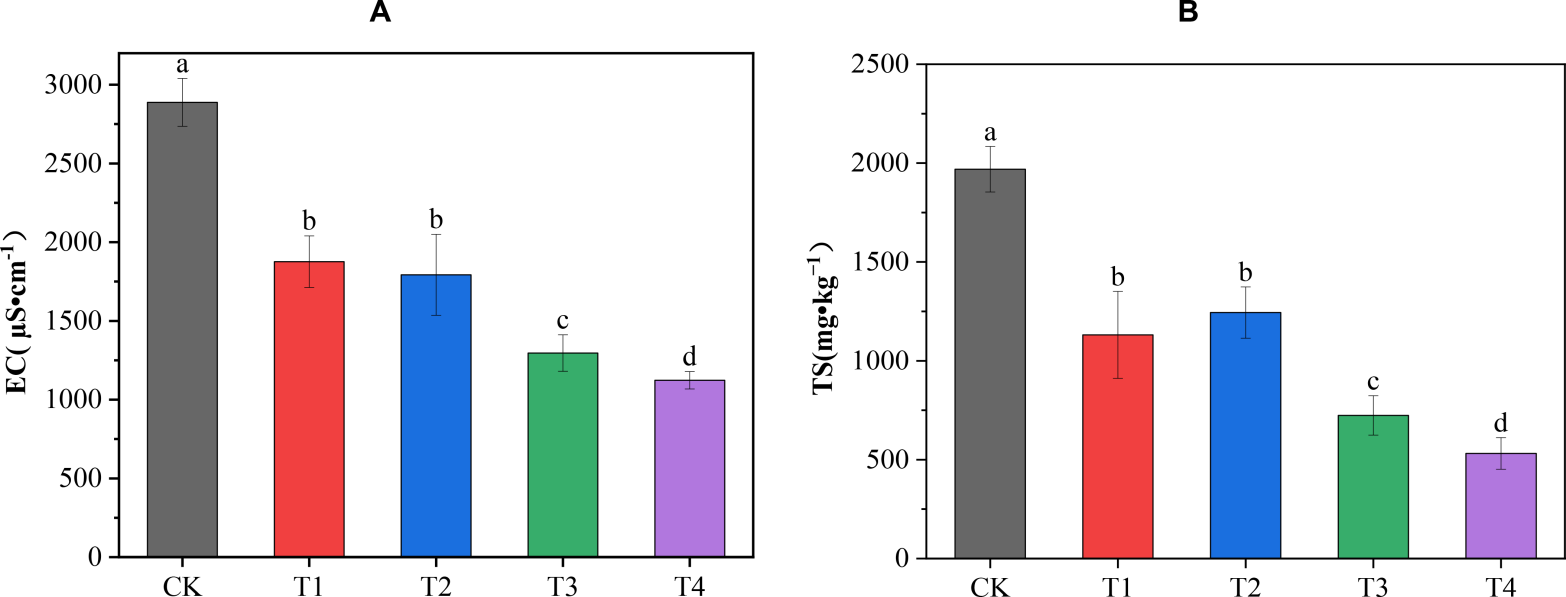
Effects of different treatments on soil EC and total salt content. **(A)** The effects of different treatments on soil EC; **(B)** the effects of different treatments on total salt (TS) content. Data are the means of six replicates (means ± standard errors). Different lowercase letters indicate significant differences between the treatments at *P* < 0.05.

All four treatments significantly reduced soil TS content and EC value compared with the CK treatment (**Figure 1**). The order of reduction effect of the four treatments on EC was T4 > T3 > T2 > T1. Compared with the CK treatment, it was significantly reduced by 61.11%, 55.12%, 37.92%, and 35.04%, respectively, among which the T4 treatment had the best reduction effect on the soil EC. The order of the reduction effect of the four treatments on TS content was T4 > T3 > T1 > T2. It was significantly reduced by 72.98%, 63.23%, 42.56%, and 36.82%, respectively, compared with the CK treatment; the T4 treatment had the best reduction effect on soil TS content. The results indicated that the reduction effects of different amounts of biochar combined with cow dung application on soil TS content and EC were better than that of biochar or cow dung application alone.

### 3.2 Soil nutrient content

Compared with the CK treatment, all four treatments significantly increased the soil nutrients, such as OM, NN, AK, and AP contents (*P* < 0.05) (**Table 2**). The four treatments had different elevating effects on soil nutrient content. The T2 treatment had the most significant effect on soil OM content (*P* < 0.05), which was significantly higher than the CK treatment by 59.93%. The T3 treatment had the most significant effect on the increase in NN content in the soil (*P* < 0.05), which was significantly higher than the CK treatment by 79.49%. The T4 treatment had the most significant effect on soil AK and AP contents (*P* < 0.05). Compared with the CK treatment, the soil nutrient content was significantly increased by 62.97% and 114.64%, respectively, indicating that biochar and cow dung application alone or combined application remarkably improved the soil nutrient content.

**Table 2:** Effects of biochar combined with cow dung application on soil nutrients.

Treatment	OM (g·kg^−1^)	NN (mg·kg^−1^)	AK (mg·kg^−1^)	AP (mg·kg^−1^)
CK	27.58 ± 0.10c	273 ± 9.53e	370 ± 5.57e	8.81 ± 0.32e
T1	36.64 ± 3.74b	297 ± 2.14d	388 ± 1.72d	11.40 ± 0.35d
T2	44.11 ± 0.32a	387 ± 1.98c	557 ± 4.20b	14.72 ± 0.26b
T3	38.74 ± 1.81b	490 ± 7.87a	476 ± 12.27c	12.91 ± 0.46c
T4	40.86 ± 2.05ab	430 ± 5.85b	603 ± 4.09a	18.91 ± 0.79a

OM, organic matter; NN, nitrate nitrogen; AK, available potassium; AP, available phosphorus. Data are the means of six replicates (means ± standard errors). Different lowercase letters indicate significant differences between the treatments at *P* < 0.05.

### 3.3 Soil exchangeable calcium and magnesium ion concentrations

**Figure 2:**
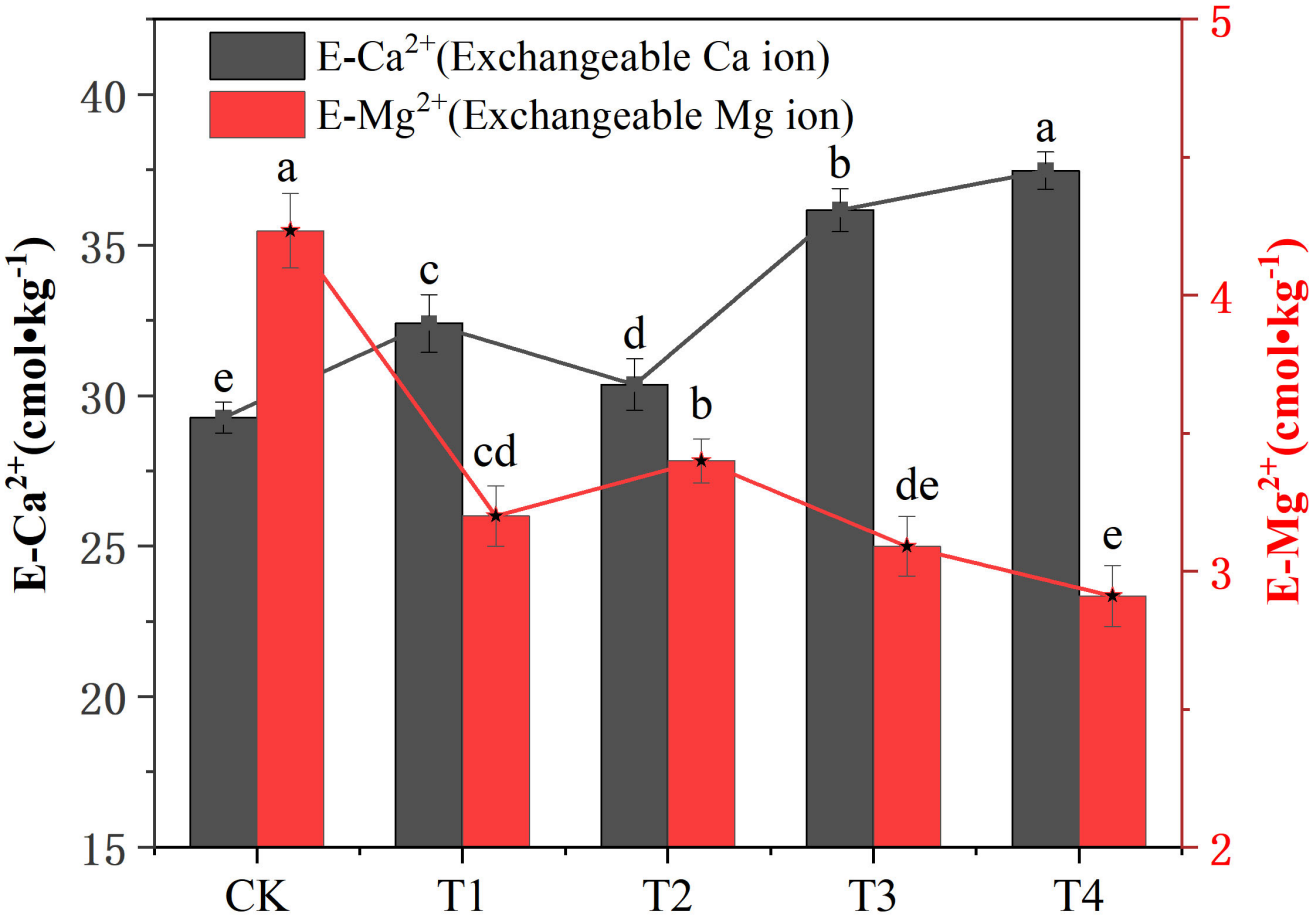
Effect of different treatments on soil exchangeable calcium (E-Ca^2+^) and magnesium (E-Mg^2+^) ion concentrations. Data are the means of six replicates (means ± standard errors). Different lowercase letters indicate significant differences between the treatments at *P* < 0.05.

Different treatments had different effects on soil E-Ca^2+^ and E-Mg^2+^ concentrations. Compared with the CK treatment, all the treatments significantly increased the concentration of E-Ca^2+^ (*P* < 0.05). The effect of the T4 treatment on the concentration of E-Ca^2+^ was the most significant, which was 26.85% higher than that of the CK treatment. All the treatments significantly reduced the concentration of E-Mg^2+^ (*P* < 0.05). The T4 treatment had the most significant effect on E-Mg^2+^ concentration, compared with the CK treatment, and it was significantly reduced by 30.72% (**Figure 2**). In addition, it can be seen from the CK treatment that the concentrations of E-Ca^2+^ and E-Mg^2+^ in the soil are quite different. Among them, the concentration of E-Mg^2+^ is higher, while the concentration of E-Ca^2+^ is lower. The application of different treatments changed the concentration of E-Ca^2+^ and E-Mg^2+^ in the soil and reduced the difference between them. Among them, the T3 treatment showed the best reduction of soil E-Ca^2+^ and E-Mg^2+^ concentration. The results showed that biochar combined with cow dung balances the concentration of exchangeable calcium and magnesium ions in soil and alleviates the degree of secondary salinization stress in soil.

### 3.4 Soil aggregate stability

All the treatments significantly increased WR0.25, MWD, and GMD of soil water-stable aggregates (*P* < 0.05) (**Table 3**). Among them, the T2 treatment had the most significant effect. Compared with the CK treatment, WR0.25 significantly increased by 14.33%, MWD significantly increased by 68%, and GMD significantly increased by 100%. However, all the treatments significantly reduced the soil structure damage rate (PAD) and soil branch dimension (D) (*P* < 0.05). Among them, the T2 and T4 treatments had the most significant effect. Compared with the CK treatment, the T2 treatment significantly reduced PAD by 55% and D by 3%. The T4 treatment significantly reduced PAD by 52% and D by 2%.

**Table 3:** Effects of biochar combined with cow dung application on the stability of soil water-stable aggregates.

Treatment	WR _0.25_ (%)	MWD (mm)	GMD (mm)	PAD (%)	D
CK	59.00 ± 1.00c	1.04 ± 0.03d	0.44 ± 0.01c	45.67. ± 0.58a	2.96 ± 0.01a
T1	70.33 ± 2.52b	1.15 ± 0.04c	0.74 ± 0.03b	24.67 ± 1.53c	2.94 ± 0.01b
T2	69.67 ± 0.58b	1.63 ± 0.05b	0.76 ± 0.04b	27.00 ± 1.00b	2.93 ± 0.01b
T3	73.33 ± 1.16a	1.75 ± 0.06a	0.88 ± 0.05a	20.67 ± 1.53d	2.87 ± 0.01d
T4	72.00 ± 1.10ab	1.58 ± 0.01b	0.79 ± 0.01b	22.00 ± 0.01d	2.90 ± 0.01c

WR0.25, water-stable aggregate content; MWD, average weight diameter; GMD, geometric mean diameter; D, soil fractal dimension. Data are the means of six replicates (means ± standard errors). Different lowercase letters indicate significant differences between the treatments at *P* < 0.05.

### 3.5 Soil enzyme activity

**Figure 3:**
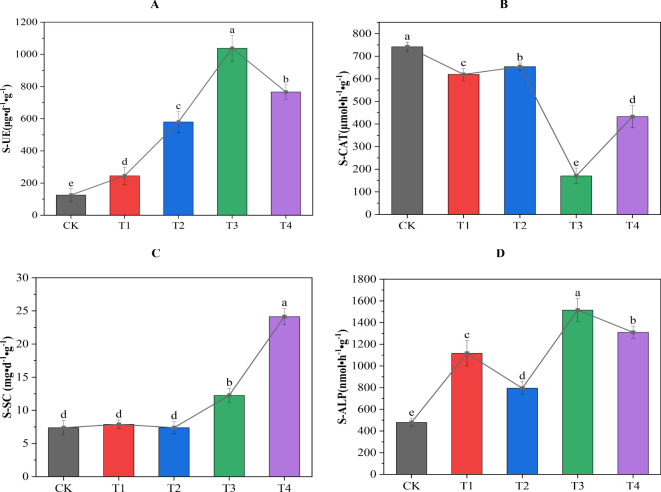
Effect of biochar combined with cow dung application on soil enzyme activity. **(A)** soil urease (S-UE); **(B)** soil catalase (S-CAT); **(C)** soil sucrase (S-SC); **(D)** soil alkaline phosphatase (S-ALP). Data are the means of six replicates (means ± standard errors). Different lowercase letters indicate significant differences between the treatments at *P* < 0.05.

It can be seen from **Figure 3** that different treatments had different effects on the enzyme activity of secondary salinized soil. All the treatments significantly increased the activity of S-UE and S-ALP. Among them, the T2 treatment had the most significant effect on the activity of S-UE and S-ALP, which was significantly higher than the CK treatment by 727% and 216%, respectively. The T4 treatment had the most significant effect on the improvement of S-SC activity, which was significantly increased by 227% compared with the CK treatment. While all the treatments significantly reduced S-CAT activity, among them, the T3 treatment had the most significant effect, as it was significantly reduced by 77% compared with the CK treatment. In summary, biochar combined with cow dung significantly increased the biological activity of urease, sucrase, and alkaline phosphatase in soil, and significantly inhibited the activity of catalase. This effect showed different intensities with an increase or decrease in the application amount. This showed that the synergistic effect of biochar and cow dung can effectively regulate and stimulate changes in soil enzyme activity under secondary salinization stress, and the synergistic effect and potential antagonistic effect between these enzymes are some of the key factors in alleviating soil secondary salinization.

### 3.6 Membrane peroxidation and antioxidant enzyme activity in leaves

**Figure 4:**
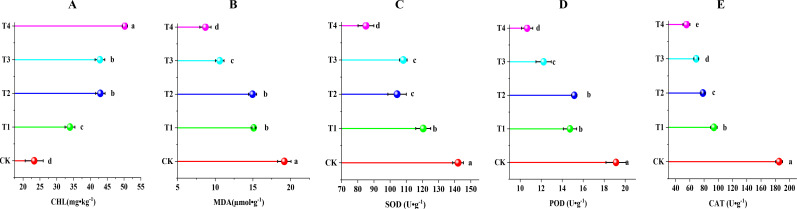
Effect of different treatments on membrane peroxidation and antioxidant enzyme activities in grapevine leaves. **(A)** chlorophyll (CHL); **(B)** malondialdehyde (MDA); **(C)** superoxide dismutase (SOD); **(D)** peroxidase (POD); and **(E)** catalase(CAT). Data are the means of six replicates (means ± standard errors). Different lowercase letters indicate significant differences between the treatments at *P* < 0.05.

As shown in **Figure 4**, compared with the control group, all the treatments significantly increased CHL content in grape leaves and significantly reduced MDA content and activities of SOD, POD, and CAT in leaves. The T4 treatment had the most significant effect on the increase in CHL content in leaves (*P* < 0.05), by 116% compared with the CK treatment. MDA content in leaves and the activities of antioxidant enzymes SOD, POD, and CAT significantly decreased (*P* < 0.05) by 54.62%, 40.15%, 44.28%, and 70.16% compared with the CK treatment as a result of the T1, T2, T3, and T4 treatments, respectively. The results showed that biochar (10 t·ha^-1^) combined with cow dung (30 t·ha^-1^) significantly reduced the damage caused by soil secondary salinization to grapes and avoided related stress.

### 3.7 Nutritional quality of grape

It can be seen in **Table 4** that the different treatments had different effects on grape quality. Among them, the T4 treatment had the most significant improvement effect on RS content and DS content, increased by 24.81% and 26.68% compared with the CK treatment, respectively. Furthermore, the decrease in the TA content was the most significant, with a significant decrease of 22.83%. In addition, the T3 treatment significantly increased the VC content by 45.66% compared with the CK treatment. The results showed that biochar combined with cow dung improved grape quality and increased economic benefit.

**Table 4:** Effects of biochar combined with cow dung application on grape quality.

Treatment	RS (g·L^-1^)	DS (%)	TA (g·L^-1^)	VC (mg·100g^-1^)
CK	296.72 ± 18.52d	13.49 ± 0.58d	9.11 ± 0.21a	3.11 ± 0.09c
T1	299.42 ± 20.74d	15.61 ± 0.41b	8.4 ± 0.22b	3.68 ± 0.11b
T2	323.45 ± 5.14c	14.44 ± 0.62cd	7.64 ± 0.27c	3.81 ± 0.21b
T3	333.73 ± 15.54b	15.2 ± 0.14bc	8.28 ± 0.16b	4.53 ± 0.25a
T4	370.35 ± 14.78a	17.09 ± 0.94a	7.03 ± 0.55d	3.96 ± 0.07b

RS, reducing sugar content; DS, soluble solid; TA, organic acid content; VC, Vitamin C content. Data are the means of six replicates (means ± standard errors). Different lowercase letters indicate significant differences between the treatments at *P* < 0.05.

### 3.8 Correlation analysis

**Figure 5:**
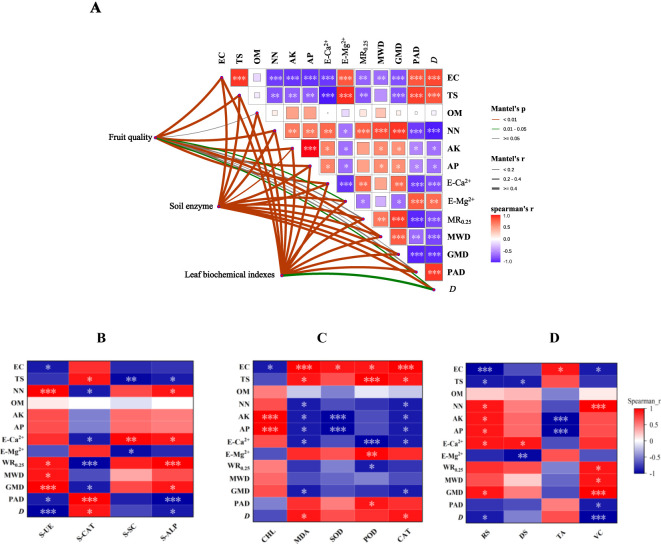
Correlation analysis. **(A)** Mantel analysis of grape growth and soil physical and chemical properties; **(B)** Spearman’s correlation analysis between soil enzyme activity and soil physical and chemical properties; **(C)** Spearman’s correlation analysis of oxidative stress factors in leaves and soil physical and chemical properties; **(D)** Spearman’s correlation analysis between grape quality and soil physical and chemical properties. Note: The thickness of the line represents the related r value of the Mantel analysis, the line color represents the Mantel correlation P-value, and the heat map color represents Spearman’s correlation coefficient, which is expressed by numerical values. The significant differences in the Spearman’s correlation analyses are marked with *, **, and *** at the levels of *P*<0.05, *P*<0.01, and *P*<0.001, respectively.

Mantel correlation analysis showed that there was a high correlation between soil enzyme activity, biochemical indexes of grape leaves, grape quality, and soil physical and chemical properties. The physical and chemical properties of secondary salinized soil are the key factors that determine the healthy growth of grapes (**Figure 5A**). Spearman’s correlation analysis of soil enzyme activity and soil physical and chemical properties showed that S-UE, S-SC, and S-ALP were negatively correlated with EC, TS, E-Mg^2+^, PAD, and D, and positively correlated with NN, E-Ca^2+^, WR_0.25_, MWD, and GMD (**Figure 5B**). Spearman’s correlation analysis between the biochemical indexes of grape leaves and soil physical and chemical properties showed that MDA, SOD, POD, and CAT were positively correlated with EC, TS, E-Mg^2+^, PAD, and D, and negatively correlated with NN, AK, AP, E-Ca^2+^, WR_0.25_, MWD, and GMD (**Figure 5C**). Spearman’s correlation analysis between grape quality and soil physical and chemical properties showed that RS, DS, and VC were negatively correlated with EC, TS, E-Mg^2+^, PAD, and D, and negatively correlated with NN, AK, AP, E-Ca^2+^, WR_0.25_, MWD, and GMD (**Figure 5D**).

## 4 Discussion

### 4.1 Improvement effect of biochar combined with cow dung application on secondary salinized soil

The accumulation of soluble salts such as sodium chloride in the soil surface layer is a significant feature of salinized soil. Soil EC is one of the key indicators of soluble salt content in soil. The higher the content of soluble salt and the corresponding EC value in the soil, the greater the osmotic pressure of the leachate. High EC values can cause osmotic stress, which in turn causes plants to suffer salt damage (Tian et al., 2024). In most environments, soil EC is positively correlated with TS (Huan et al., 2008; Rivera-García et al., 2022; Tian et al., 2024). Corresponding conclusions were also drawn through the correlation analysis in this experiment (**Figure 5A**). This study showed that applying biochar and cow dung, either alone or in combination, reduced secondary salinization, which was consistent with previous research findings. Fermented cow dung and biochar as soil amendments can improve the physical and chemical properties of saline-alkali soil, reduce salinity and pH value, and alleviate salt stress (Li et al., 2022a; Pan et al., 2022; Wang et al., 2023b). This may be related to the nature of cow dung and biochar itself; organic manure can improve soil water conductivity, improve the efficiency of soil leaching, and reduce secondary salinization (Kayode Steven et al., 2018). Biochar has a huge specific surface area and strong adsorption capacity for water-soluble cations in soil, thus reducing the soil EC (Duan et al., 2021a). This study also showed that the combined application of biochar and cow dung had the most significant improvement effect on soil secondary salinization compared with a single application; the improvement effect of halving combined application was weaker than that of full-dose combined application. This may be due to the excessive application of the two replacing parts of the undisturbed soil and changing the soil particle size structure, which is conducive to the leaching of base ions, thereby reducing the TS in soil and EC. This study showed that the application of biochar significantly improved soil nutrient content, but this effect was worse than that of other treatments (**Table 2**). This may be related to the nutrient content in intensive agricultural production, where the application of biochar promotes the release of nutrients from the soil. In soils modified with biochar, typically higher C/N may result in N fixation (Wang et al., 2019), especially the fixation of NN (Manolikaki and Diamadopoulos, 2019). The application of the biochar did not show a fixation effect on NN. Excessive NN negatively impacts the ecological environment and human production and daily life; the accumulation of NN causes secondary salinization of soil and aggravates soil pressure (Yang et al., 2024). Cow dung contains nutrients available to plants (nitrate, phosphate, calcium, and potassium) and has high porosity, good aeration, drainage, and water retention (Joshi et al., 2015; Font-Palma, 2019). On the one hand, the combined application of the two can improve the secondary salinized soil; on the other hand, it can improve the availability of soil nutrients and promote the release of nutrients in the soil. Exchangeable calcium and magnesium are the main exchangeable base ions in soil. The content of exchangeable calcium and magnesium in soil directly reflects the ability of the soil to supply calcium and magnesium (Sharma et al., 2018). This study showed that the combined application of biochar and cow dung significantly reduced the exchangeable calcium content and significantly increased the exchangeable magnesium content in soil. However, the combined application of biochar and cow dung had opposite effects on soil exchangeable calcium and magnesium (**Figure 2**). Sardans et al. (2023) showed that Ca^2+^ content gradually increased with the extension of repair time and the decrease in Mg^2+^ content also promoted the gradual increase in Ca^2+^ content. With the extension of time, the content of Mg^2+^ in the soil decreased continuously and was negatively correlated with the repair time. This is related to the increase in Ca^2+^ and Mg^2+^ contents, which antagonistically affect Mg content. With the increase in Ca^2+^ content, the activity of Ca^2+^ in the soil is inhibited, which is not conducive to the absorption of Mg by plants, However, too high Mg^2+^ levels will also produce salt damage (Ying et al., 2022). This study also showed that biochar combined with cow dung increased soil water-stable aggregates and reduced soil structure damage rate and soil branch dimension. The possible reason is that divalent cations (Ca^2+^ and Mg^2+^, but especially Ca^2+^) in soil can enhance the cementation between soil particles and colloids, such as humus, and promote the formation and stability of aggregates due to their strong ion bridge (Totsche et al., 2018) and cation polarization (Huang et al., 2016; Ishiguro and Nakajima, 2000).

### 4.2 Effects of biochar combined with cow dung application on grape leaf stress

The Mantel correlation analysis showed that the antioxidant enzyme activity of grape leaves was significantly positively correlated with soil TS content and EC value (**Figure 5C**), the higher the soil TS content and EC value, the stronger the antioxidant enzyme activity of the grape leaves. Previous studies have shown that under salt stress, plants produce a large amount of ROS, such as O_2_⁻, H_2_O_2_, and OH⁻ (Chen et al., 2015). These ROS can cause oxidative stress and destroy intracellular proteins, DNA and cellular lipids, and other biological macromolecules, resulting in damage to cell structure and function. In order to cope with this oxidative stress, plants have evolved a complex antioxidant system, including SOD, CAT, and POD and other antioxidant enzymes (Li et al., 2022b; Yu et al., 2022; Iwaniuk et al., 2023). SOD is the first line of defense in the antioxidant enzyme system, which can disproportionate O_2_⁻ to H_2_O_2_ and O_2_, thereby reducing the toxicity of O₂⁻ (Sgherri et al., 2013). CAT and POD are mainly responsible for scavenging H_2_O_2_. CAT catalyzes the decomposition of H_2_O_2_ to H_2_O and O_2_, while POD uses H_2_O_2_ as an electron acceptor to oxidize other substrates, thereby indirectly scavenging H_2_O_2_ (Samiksha et al., 2016). Under salt stress, the ROS content in plants increases, resulting in increased antioxidant enzyme activity to remove excessive ROS. However, this increase in activity is a stress response of plants to stress. Long-term high activity of antioxidant enzymes will consume energy and resources in plants and have a negative impact on plant growth and development (Gill and Tuteja, 2010; Waszczak et al., 2018). In this study, the application of biochar and cow dung significantly reduced the TS content and EC value of the soil, thereby alleviating the salt stress of plants. By reducing salt in the soil, biochar and cow dung reduced the production of ROS in the plants, thereby reducing the demand for antioxidases. Under reduced salt stress, the activity of antioxidant enzymes in plants decreased accordingly. The decreased activity of SOD, POD, and CAT means that the plants no longer need to consume excessive energy to maintain a high level of antioxidant defense, thereby reducing the oxidative damage caused by ROS. In addition, with the decrease of salt stress, the content of CHL in plants increases, which helps to improve the efficiency of photosynthesis and promote the growth and development of plants (Moustaka and Moustakas, 2023). MDA is the main product of membrane lipid peroxidation. A decrease in MDA content indicates that the integrity of the plant cell membrane is protected and the cell structure and function are more stable (Hasanuzzaman et al., 2021). These studies fully show that plants will resist adversity through various physiological and biochemical reactions under adversity, and achieve their own healthy growth and development. Therefore, the combined application of biochar and cow dung can effectively reduce the oxidative damage caused by salinity and improve the quality of grapes by reducing soil salinity, reducing ROS content in plants, and regulating antioxidant enzyme activities. This physiological improvement helps grapes to maintain growth under adversity and reduce yield loss. The improvement of grape yield and quality directly affects market competitiveness. High-quality grapes attract more consumers, improve the market value of products, and increase the economic benefits for farmers. At the same time, by reducing the losses caused by stress, farmers can reduce production costs and improve economic efficiency.

## 5 Conclusions

In conclusion, biochar combined with cow dung application stimulated an increase in soil-related enzyme activities by coordinating and balancing the exchangeable calcium and magnesium ions in the soil and increased the water-stable aggregates to reduce the salt content in the soil. In addition, biochar combined with cow dung application alleviated the stress of secondary salinization on the growth of grape plants, and reduced the oxidative stress response of leaves, and ultimately improved the quality of grape berries due to improved soil quality. From the perspective of economic benefits and sustainable development of intensive planting, 5 t·ha^-1^ biochar combined with 15 t·ha^-1^ cow dung is more suitable for viticulture to prevent the excessive application of farm manure and ensure the sustainable development of grape-producing areas. After a 3-year trial period, biochar combined with cow dung has a positive impact on soil health. Based on the existing research, they are safe for the growth of grape plants and may have a positive effect on their ability to resist diseases and pests. It is recommended that policymakers promote this soil improvement method to farmers and encourage its use through subsidies and technical support to achieve a win-win situation with both soil and economic benefits. The improved technology has wide applicability globally, especially in areas facing similar soil problems, and can help to enhance the sustainability of global agriculture and contribute to global food security and eco-agricultural development.

## Data Availability

The original contributions presented in the study are included in the article/supplementary material. Further inquiries can be directed to the corresponding authors.
